# Evaluating the Effects of High RAP Content and Rejuvenating Agents on Fatigue Performance of Fine Aggregate Matrix through DMA Flexural Bending Test

**DOI:** 10.3390/ma12091508

**Published:** 2019-05-09

**Authors:** Chenchen Zhang, Qi Ren, Zhendong Qian, Xudong Wang

**Affiliations:** 1 Intelligent Transport System Research Center, Southeast University, Nanjing 210096, China; 230129418@seu.edu.cn; 2 Research Institute of Highway Ministry of Transport, Beijing 100088, China; xd.wang@rioh.cn; 3 Jiangsu Transportation Research Institute, Nanjing 210017, China; qrendavis@gmail.com

**Keywords:** reclaimed asphalt pavement, fatigue, linear amplitude sweep, fine aggregate matrix, flexural bending, viscoelastic continuum damage, rejuvenating agent

## Abstract

High percentage reclaimed asphalt pavement (RAP) is prevailing in pavement engineering for its advantages in sustainability and environmental friendliness, however, its fatigue resistance remains a major concern. Fine aggregate matrix (FAM) is a crucial part in the fatigue resistance of asphalt mixtures with high RAP content. Hence, the linear amplitude sweep (LAS) test of FAM has been developed to study the fatigue resistance of asphalt mixtures. However, the torsional loading mode of the LAS test with a dynamic shear rheometer (DSR) is a limitation to simulate traffic load. In this paper, an alternative LAS test for FAM with high RAP content was proposed. Beam FAM specimens were tested using a dual-cantilever flexural loading fixture in a dynamic mechanical analyzer (DMA). To investigate the influence of RAP content and the rejuvenating agent (RA), four kinds of FAM mixes were tested with this method to study their fatigue resistance. The test results suggested that the repeatability of this alternative approach was reliable. A fatigue failure criterion based on maximum C×N was defined. Then, fatigue life prediction models based on viscoelastic continuum damage (VECD) analysis were established according to the LAS test results and validated by a strain-controlled time sweep (TS) test. It turned out that as RAP content increased, the modulus of FAM would be significantly raised, accompanied with a drop in the phase angle. The fatigue life of FAM would be greatly shortened when the RAP binder replacement rate reached 50%. Adding RA could considerably improve the dynamic properties of FAM mixes with high RAP content, resulting in a decrease in modulus, increase in phase angle and elongating fatigue life, but could not recover to the level of virgin binder.

## 1. Introduction

Reclaimed asphalt pavement (RAP) technology is an ideal and widely used sustainable technology in pavement engineering. RAP showed significant advantages against traditional hot mix asphalt (HMA) pavement, including non-renewable natural resources preservation, relief of landfill pressure, reducing energy consumption and greenhouse gas emissions [1]. As RAP content increases from 15% to 40% in HMA, pavement cost will be cut by $3.40 to $6.80 per ton [2]. Based on life cycle assessment (LCA), when RAP content rises from 30% to 50%, the energy consumption can be reduced by 16% to 25% [3]. Massive RAP is generated during pavement maintenance and rehabilitation all cross the world. According to the National Asphalt Pavement Association (NAPA), 76.2 million tons of RAP were reclaimed in the United States in 2017 [4]; in all European Union member countries, a total amount of 50 million tons of RAP were reclaimed in 2015 [5]; in China, this number is over 60 million tons in recent years [6]. Researchers are working on further raising RAP content, from 20–30% to 40–50%, even up to 100% [7], in order to achieve better sustainability in pavement construction. Studies have shown that HMA will become stiffer and more brittle with higher RAP content because aged binder exists in the mixture. Aged binder can improve the permanent deformation resistance, but also has a negative effect on the fatigue resistance of pavement [8]. To mitigate stiffness and improve fatigue cracking resistance, rejuvenating agent (RA) is commonly used in HMA with high RAP content [9].

In order to study the fatigue performance of asphalt pavement with RAP, several classical laboratory fatigue testing methods have been developed. Since the aged binder in RAP is considered as the main contributor to the fatigue life, the majority of researchers choose to study its fatigue properties through a method known as extraction-recovery [10]. However, this approach is not preferred by researchers for many reasons. The chemical reactions between bitumen and solvent can make the binder stiffer; studies have also proved that even a small amount of residual solvent can significantly alter the rheologic properties of the binder [11]. Meanwhile, the aged and virgin binders will be totally blended, which can camouflage the actual blending level in HMA [12]. Besides, asphalt solvent is also considered to be harmful to operators and may cause hazardous waste disposal issues.

The classical four-point beam test is another common approach to study the fatigue resistance of HMA. This test has been widely used by researchers and proved to be one of the most effective approaches in fatigue study. The only flaw is that four-point beam fatigue test is quite expensive and time-consuming. In addition, the repeatability of fatigue tests on full-graded HMA is not good due to the complexity and heterogeneity of materials [13]. A solution to solve the repeatability problem is to test the fine aggregated matrix (FAM) instead of full-graded HMA [14]. FAM is a mixture of binder, fillers and fine aggregates, which is supposed to have better homogeneity than coarse aggregate particles. Micro-cracking in HMA is usually considered to initiate and propagate in the FAM phase, so studying the fatigue resistance of FAM can help better understand the mechanism of fatigue cracking [15]. The linear amplitude sweep (LAS) test of cylindrical specimens was developed to study the fatigue performance of FAM mixes. A solid torsion bar fixture is used to fix the specimen in dynamic shear rheometer (DSR) [16]. Then, a strained-controlled oscillatory shear is applied to the FAM cylinder. Since the amplitude of strain is increasing linearly to accelerate internal damage, this method is called the linear amplitude sweep test. According to LAS test results, a mathematical model to predict the fatigue lives of FAMs can be established based on viscoelastic continuum damage (VECD) analysis, which can effectively distinguish the fatigue performance of different FAM mixes [17]. Compared with the traditional four-point beam fatigue test, the LAS test exhibits obvious advantages, such as time efficiency, repeatability and simplicity, which made it a prevalent test method for fatigue characterization [18]. However, complex viscoelasticity and anisotropic behaviors can be observed in asphalt materials, which means its mechanical behavior would be different under different loading modes. The LAS test with DSR applies torsional load on FAM specimens, while the fatigue of asphalt pavement is generally subjected to flexural bending caused by traffic load, which are two totally different loading modes. To address this issue, a LAS test of FAM mixes under flexural bending should be developed. In the field of polymer study, a solid dual-cantilever flexural loading fixture has been successfully applied to characterize the dynamic behavior of materials [19]. Since the viscoelasticity of FAM mixes is similar to polymer materials, the same fixture is employed to study the fatigue resistance of FAM with high RAP content.

The major objective of this study is to study the influence of RAP content on FAM mixes. A flexural bending LAS test was employed to study the fatigue performance. In detail, the following tasks need to be accomplished:

1. Assess the LAS test method for beam FAM specimens under flexural bending mode which serves as an alternative approach for the torsional LAS test with DSR.
2. Define a reasonable failure criterion of FAM mixes for LAS test under flexural bending mode.
3. Establish fatigue life prediction models based on VECD analysis according to LAS tests, and validate the models with measured data from time sweep (TS) tests.
4. Study the influence of high RAP content (over 25%) on the fatigue lives of FAMs and evaluate the effectiveness of rejuvenating agent.

## 2. Materials and Test Procedures

### 2.1. Materials

In this study, a typical virgin bituminous binder AH-70 with 60/80 penetration grade was selected, which has been widely used in heavy traffic freeways in China. Table 1 lists the technical index of the virgin binder. Limestone was used as the virgin aggregate. RAP passing through a 2.36 mm sieve was supplied by a local asphalt mixing plant in Beijing. A rejuvenating agent (RA) based on petroleum technology was used in this study. According to the recommendations of the producer, the RA content was 10% of the total weight of asphalt.

**Figure 1:**
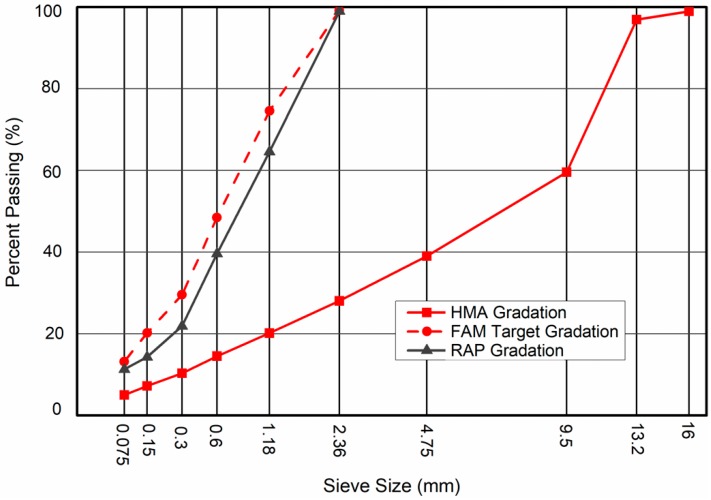
Gradations of hot mix asphalt (HMA), fine aggregate matrix (FAM) and reclaimed asphalt pavement (RAP).

The maximum aggregate size of the FAM was 2.36 mm in this study. The target gradation and binder content of FAM mixes were determined by solvent extraction of the HMA fine portion (<2.36 mm), which was suggested by Yuan et al. [20]. This procedure aimed to ensure that the FAM mixes could represent the fine portion of HMA. The binder content of FAM and RAP were 9.0% and 7.3%, respectively. Figure 1 shows the gradations of HMA, FAM and RAP.

Four FAM mixes were tested as summarized in Table 2. In the first group, FAM mixes were batched with virgin binder, which served as the control group. In the second group, a 25% RAP binder replacement was applied, which is commonly used in maintenance and rehabilitation. In the third group, this rate was raised to 50% to examine the influence of RAP content at higher levels. In the last group, the RAP binder replacement rate was still 50%, which was consistent with the third group, but RA was added into the FAM to assess its effect. For all four FAM mixes, the gradation was kept the same with the target FAM gradation, no matter what the RAP and RA contents were. The virgin binder and aggregate contents were adjusted according to RAP binder replacement rates in order to match the target FAM gradation.

### 2.2. FAM Specimen Preparation

**Figure 2:**
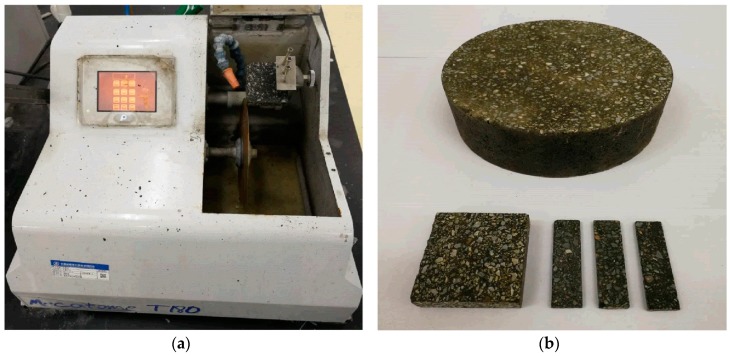
FAM specimen preparation: (**a**) precision cutting machinery; (**b**) FAM specimens.

FAM cylindrical specimens with 150 mm diameter and 50 mm height were fabricated using a Superpave Gyratory Compactor. The target air void content of cylindrical specimens was 6%. The FAM cylinders were cut by a Presi MECATOME precision cutting instrument (Figure 2a) to 60 mm × 45 mm × 15 mm rectangles, then sliced to 60 mm × 15 mm × 3.5 mm beam specimens. Since the passing percentage of the 2.36 mm sieve is 100% for FAM specimens, a thickness of 3.5 mm was considered enough to avoid the scale effect for most of the aggregates smaller than 1.18 mm. Figure 2b shows the compacted cylindrical specimen, rectangular specimen and beam specimens.

### 2.3. Test Setup and Procedures

**Figure 3:**
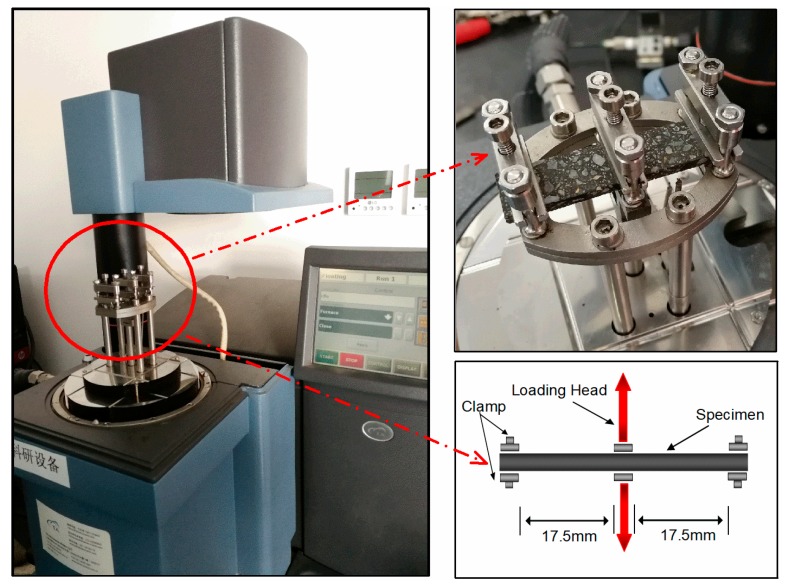
Dynamic mechanical analyzer (DMA) dual-cantilever bending fixture.

A TA Instruments dynamic mechanical analyzer (DMA) Q800 apparatus (TA Instruments, New Castle, DE, USA) was used in this study along with a dual-cantilever flexural bending fixture. The FAM specimen was gripped by two clamps at its ends, then a movable head could apply load in the center of the beam, as illustrated in Figure 3. In order to examine the repeatability of the tests, three replicate tests were conducted for each group of FAM mixes.

#### 2.3.1. Frequency Sweep Test

To evaluate the undamaged dynamic properties of FAMs within the linear viscoelastic (LVE) region, a frequency sweep test was conducted to test the dynamic modulus and phase angle. The test temperature was 20 °C and loading frequencies ranged from 0.1 Hz to 25 Hz. A viscoelastic parameter m is indispensable to characterize damage property in VECD analysis, which is defined as the slope of the master curve (dynamic modulus versus loading frequency on log–log diagram) in the LVE region [21]. Recent studies have investigated the LVE region of similar FAM mixes and suggested that a strain level of 0.002% would be small enough to ensure that FAMs remained undamaged during the test [17,20]. Therefore, the constant amplitude strain of frequency sweep test was set to 0.002%.

#### 2.3.2. Linear Amplitude Sweep Test

**Figure 4:**
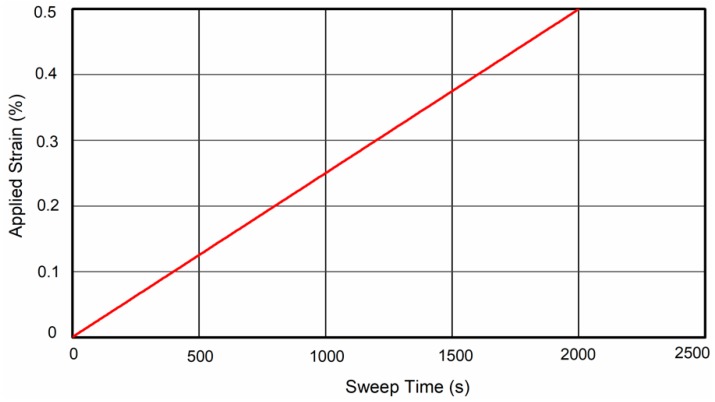
Strain applied in the linear amplitude sweep (LAS) test.

In the LAS test, the testing temperature was 20 °C and loading frequency was 10 Hz. The applied strain was linearly increased from 0.001% to 0.5% in 2000 s, as shown in Figure 4.

The LAS test can be treated as an approach to accelerate fatigue damage. Combined with VECD analysis, its results can be used to efficiently determine the regression parameters for the traditional fatigue equation, which is commonly used in strained-controlled fatigue tests. The relationship between fatigue life $N_{f}$ and applied strain amplitude $\varepsilon_{p}$ can be expressed as:

(1) $$N_{f}=A{\left(\varepsilon_{p}\right)}^{-B}$$

According to Shapery’s work potential theory [22], the damage intensity S of viscoelastic materials can be expressed in terms of the work performed $W^{R}$:

(2) $$\frac{dS}{dt}={\left(-\frac{\partial W^{R}}{\partial S}\right)}^{\alpha }$$

where: t is the time, s; α is equal to 1+1/m [23].

According to elastic–viscoelastic correspondence principle [24], the flexural pseudo strain $\varepsilon_{}^{R}$ can be defined as follows:

(3) $$\varepsilon_{}^{R}=\frac{1}{E_{R}}\int_{0}^{t}E(t-\xi )\frac{\partial \varepsilon }{\partial \xi }d\xi$$

where: $E_{R}$ is a constant reference modulus, usually selected as 1; E(t) is LVE relaxation modulus, MPa; ε is the flexural strain, percentage; ξ is an integral variable. The flexural LVE stress $\sigma_{LVE}$ in MPa can be expressed as:

(4) $$\sigma_{LVE}=\int_{0}^{t}E(t-\xi )\frac{\partial \varepsilon }{\partial \xi }d\xi$$

Combining Equations (3) and (4), the flexural LVE stress $\sigma_{LVE}$ can be rewritten as:

(5) $$\sigma_{LVE}=E_{R}\varepsilon^{R}$$

In order to quantify material integrity, pseudo stiffness C(S) can be defined as a damage variable, as follows:

(6) $$C\left(S\right)=\frac{\sigma }{\sigma_{LVE}}=\frac{\sigma }{\varepsilon^{R}}$$

where: σ is the flexural stress, MPa. For viscoelastic materials under periodical loading, the pseudo flexural strain amplitude $\varepsilon_{Pi}^{R}$ in percentage and pseudo stiffness C(S) in cycle *i* can be separately rewritten as:

(7) $$\varepsilon_{Pi}^{R}=\frac{1}{E_{R}}\varepsilon_{Pi}^{}\left|E_{LVE}^{*}\right|$$

(8) $$C\left(S\right)=\frac{\sigma_{Pi}}{\varepsilon_{Pi}^{R}}$$

where: $\varepsilon_{Pi}^{}$ is the flexural strain amplitude in cycle *i*, percentage; $\left|E_{LVE}^{*}\right|$ is the flexural dynamic modulus in the LVE region, MPa; $\sigma_{Pi}$ is the flexural stress amplitude in cycle *i*, MPa.

The work performed $W^{R}$ in Equation (2) can be quantified using pseudo strain energy density [25], as follows:

(9) $$W^{R}=\frac{1}{2}C\left(S\right){\left(\varepsilon_{Pi}^{R}\right)}^{2}$$

Combining Equations (2), (7), (8) and (9) and integrating with a numerical approach, the damage intensity can be written as a function of time t:

(10) $$S\left(t\right)≅{\sum_{i=1}^{n}\left[\frac{1}{2}\left(C_{i-1}-C_{i}\right){\left(\varepsilon_{Pi}^{R}\right)}^{2}\right]}^{\frac{\alpha }{1+\alpha }}{\left(t_{i}-t_{i-1}\right)}^{\frac{1}{1+\alpha }}$$

where: n is the number of loading cycles. The curve of pseudo stiffness C(S) versus damage intensity S typically follows a power model as follows [15]:

(11) $$C(S)=1-C_{1}{\left(S\right)}^{C_{2}}$$

where: $C_{1}$, $C_{2}$ are regression coefficients.

Finally, by combining Equations (2), (7), (9) and (11) and integrating, the fatigue life prediction model can be described as [15]:

(12) $$N_{f}=\frac{f{\left(S_{f}\right)}^{1+\alpha \left(1-C_{2}\right)}}{\left[1+\alpha (1-C_{2})\right]{\left(0.5C_{1}C_{2}\right)}^{\alpha }{\left(\left|E_{LVE}^{*}\right|\right)}^{2\alpha }}{\left(\varepsilon_{p}\right)}^{-2\alpha }$$

where: f is the loading frequency, Hz; $S_{f}$ is the damage intensity S at failure point. The fatigue model coefficients *A* and *B* can be written as:

(13) $$A=\frac{f{\left(S_{f}\right)}^{1+\alpha \left(1-C_{2}\right)}}{\left[1+\alpha (1-C_{2})\right]{\left(0.5C_{1}C_{2}\right)}^{\alpha }{\left(\left|E_{LVE}^{*}\right|\right)}^{2\alpha }}$$

(14) B=2α

#### 2.3.3. Time Sweep Test

To validate the fatigue life prediction model above, strain-controlled TS tests were employed. Four strain levels (0.07%, 0.08%, 0.09% and 0.1%) were selected with no rest period. The testing temperature and loading frequency of the TS test was consistent with the LAS test (20 °C, 10 Hz).

## 3. Results and Discussion

### 3.1. Frequency Sweep Test

**Figure 5:**
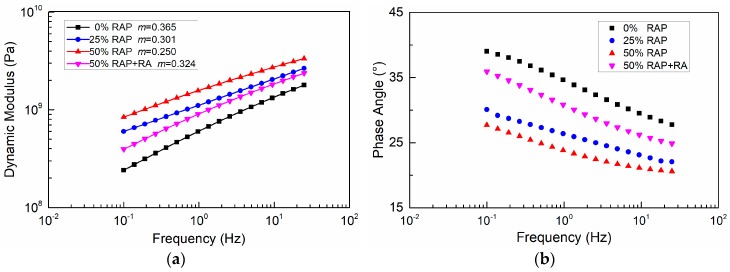
Frequency sweep results of FAM mixes: (**a**) Dynamic modulus; (**b**) Phase angle.

The frequency sweep test results of four FAM mixes are illustrated in Figure 5. The dynamic modulus and phase angle exhibited significant relativities with loading frequency. When RA was not present in FAM mixes, the dynamic modulus would increase with the RAP content, especially at lower loading frequencies. At 0.1 Hz, when the RAP binder replacement rate was 25% and 50%, the dynamic modulus of FAM mixes was increased by approximately 1.5 and 2.5 times compared with virgin binder, respectively. However, once RA was added, FAM mixes were softened which resulted in a drastic drop of modulus. Phase angle displayed a reverse trend of dynamic modulus, which also implied the effect of RAP and RA. Furthermore, in the linear viscoelastic region, the dynamic modulus showed a linear relationship with loading frequency on a log–log scale as expected. It can be inferred from Figure 5a that the slope m was smaller for FAMs with a higher modulus.

### 3.2. Failure Criterion Definition of LAS and TS Test

The fatigue process of materials starts with the presence of invisible internal microcracks, and then those microcracks gradually grow up and propagate to macro fractures under repeated loading. Failure criterion defines the critical point from stable to unstable damage growth stage. A reasonable criterion is a necessary part of fatigue life prediction models.

**Figure 6:**
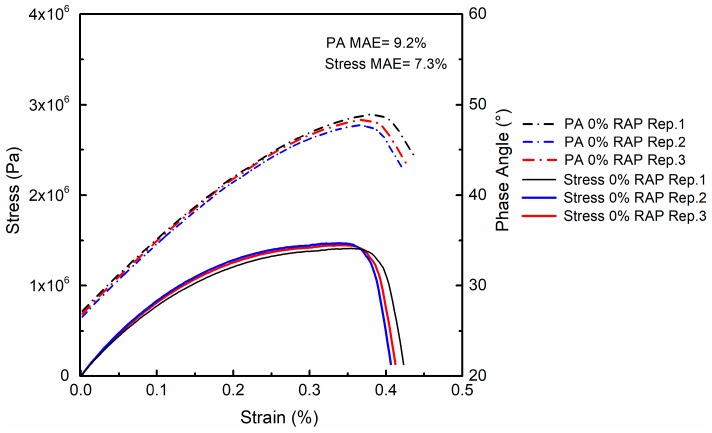
Typical replicate results of LAS test (0% RAP). MAE = mean absolute error.

**Figure 7:**
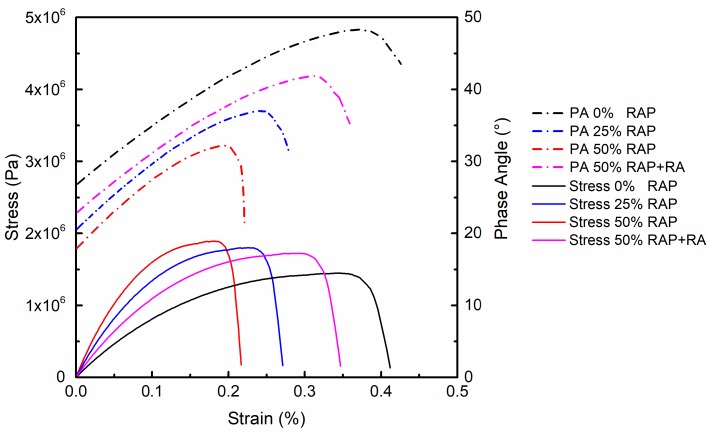
LAS test results of FAM mixes.

As an illustration, Figure 6 exhibits the replicate LAS test results of FAM mixes with virgin binder. Well-repeatable behaviors were observed for both stress–strain and phase angle–strain curves, with a mean absolute error (MAE) of 9.2% and 7.3%, respectively. The LAS test results of all four groups of FAM mixes are plotted in Figure 7. Stress and phase angle kept increasing with strain until a critical peak point was reached. The corresponding strains for peak stress and peak phase angle were close, but phase angle reached its peak slightly slower than stress. The maximum stress could be regarded as a yielding point, beyond which the specimen could not stand more loading. After the specimen yielded, phase angle started to drop, which means the fatigue damage reached a limit [26]. This phenomenon followed the yield-failure pattern of materials, and the final failure of FAM specimens was represented by the drop of phase angle. Thus, the peak phase angle could be regarded as the fatigue failure criterion in LAS test, which is also a typical failure indicator extensively used in the strain-controlled TS test [27]. It can be inferred from Figure 7 that FAM mixes with higher RAP content showed higher peak stress, lower peak phase angle and lower failure strain. Once RA was added into the FAM mix with high RAP content, a significant decrease of peak stress, increase of peak phase angle and higher failure strain could be clearly observed.

**Figure 8:**
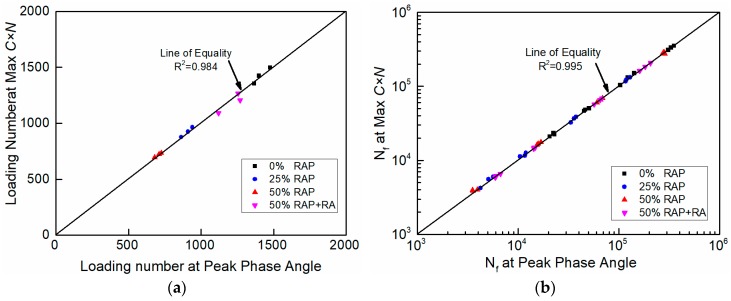
Comparison of failure criterion between peak phase angle and max *C × N*: (**a**) LAS test; (**b**) time sweep (TS) test.

Another common phenomenological definition to assess fatigue failure is the maximum C×N. C refers to the pseudo stiffness C(S) in Equation (6), indicating material integrity. N is the number of loading cycle. Maximum C×N is considered as a reasonable failure indicator in both LAS and TS test [28]. The numbers of loading cycles at peak phase angle and at maximum C×N are shown in Figure 8. Obviously, the maximum C×N and the peak phase angle appeared simultaneously in both LAS tests and strain-controlled TS tests. According to American Association of State Highway and Transportation Officials (AASHTO) T321-17, maximum S×N (S refers to stiffness) is used to define fatigue failure for traditional four-point beam fatigue test of HMA, which is a similar parameter to maximum C×N [26]. Therefore, maximum C×N was selected as the failure criterion of FAM mixes in this study to keep consistency with AASHTO standards (T321-17).

### 3.3. Fatigue Prediction Model Based on VECD Analysis

**Figure 9:**
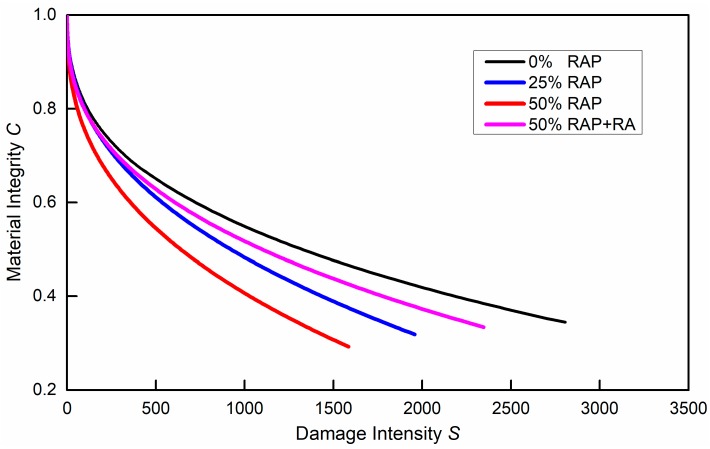
Damage characterization curves of FAM mixes from LAS tests.

Figure 9 shows the damage characteristic curves (C−S) from LAS tests. As damage intensity S increased, material integrity C gradually reduced from 1 to 0.3–0.4, which was the defined failure point at maximum C×N. A higher RAP binder replacement rate would lead to faster reduction of C compared with virgin binder. Also, the addition of RA in FAM mixes with high RAP content would substantially improve damage resistance but could not fully recover it to the level of virgin binder.

According to the C−S curve, the fatigue life prediction model could be established as Equation (12) based on VECD analysis. The regression parameters are listed in Table 3.

**Figure 10:**
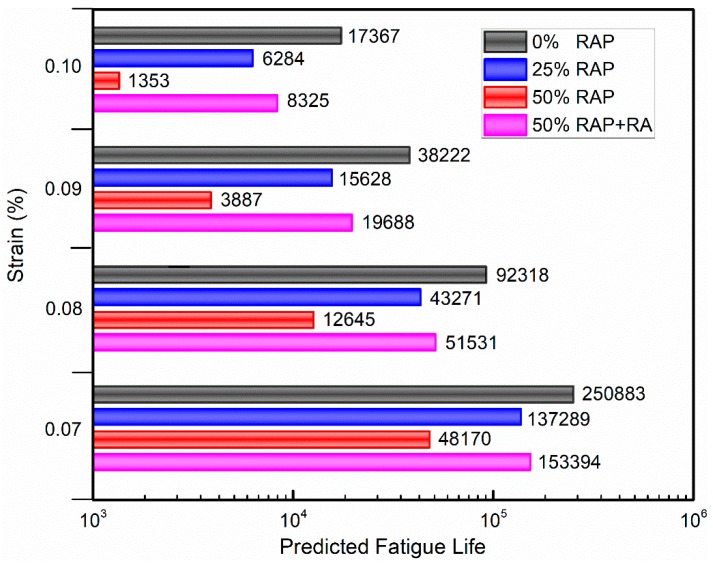
Predicted fatigue lives for FAM mixes at four strain amplitudes.

The predicted fatigue lives for FAM mixes at four different strain levels are displayed in Figure 10. As expected, a higher RAP content would negatively influence the fatigue performance, resulting in a much shorter fatigue life. Compared with virgin binder, the fatigue lives of FAMs with 50% RAP binder replacement rate were reduced by 80.8%, 86.3%, 89.8% and 92.2% at strain levels of 0.07%, 0.08%, 0.09% and 0.10%, respectively. However, when RA was added into the FAM mixes, their fatigue lives were greatly extended by 3.2, 4.1, 5.1 and 6.2 times, which were recovered to 61.1%, 55.8%, 51.5% and 47.9% of virgin binder FAM mixes, respectively. Obviously, the existence of RA in FAM mixes with high RAP contents would significantly mitigate stiffness and improve cracking resistance, especially at higher strain levels.

### 3.4. Validation of Fatigue Prediction Model from TS Test

The results of strain-controlled TS tests are listed in Table 4. The coefficients of variation ranged from 2.3% to 15.9%, which was smaller compared with fatigue tests of full-graded HMA. The TS test results suggested that the fatigue performance ranking of four groups of FAM mixes was virgin binder, 50% RAP + RA, 25% RAP and 50% RAP from best to worst, which was consistent with model predictions.

**Figure 11:**
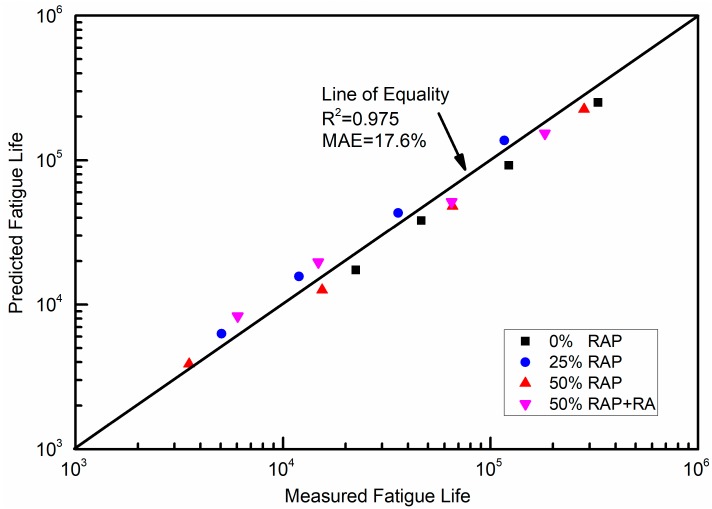
Measured fatigue lives versus predicted fatigue lives. R^2^ = correlation coefficient.

The predicted fatigue lives are plotted along with the measured results in Figure 11. Fairly good consistency with the line of equality could be observed with a correlation coefficient *R*^2^ of 0.975 and MAE of 17.6%. The fatigue life prediction models based on LAS test results are considered reasonable.

Table 5 shows the estimated efficiency of the LAS test and strain-controlled TS test. In general, to acquire the curve of fatigue life for a given FAM mix, the TS test requires 12 individual tests and will take approximate 27 h, while the LAS test only needs three tests and 3 h. Based on the estimates, the LAS test is considered more efficient than the TS test.

## 4. Conclusions

In this study, a flexural bending test method using DMA for FAM mixes with high RAP content was proposed. Four groups of FAM mixes were tested with this method to investigate the impact of RAP content on the fatigue properties and effectiveness of RA. The following conclusions are made:

1. As an alternative test method for torsion bar test with a DSR, the LAS test of FAM mixes under flexural bending mode can provide acceptable data with good repeatability.
2. The phase angle peak and the maximum appeared simultaneously in both LAS tests and strain-controlled TS tests. In this study, the maximum was selected as a reasonable parameter for defining fatigue failure criterions.
3. Based on the maximum failure criterion and VECD analysis, fatigue life prediction models can effectively capture the fatigue resistance of different FAMs. The predicted fatigue lives were well-consistent with the measured results of TS tests.
4. Higher RAP content will considerably increase the stiffness of FAM mixes, resulting in a decrease in phase angle and fatigue resistance. The presence of petroleum-based rejuvenating agents will soften FAMs, resulting in a significant recovery of the lost fatigue resistance.

To conclude, the LAS test under flexural bending mode is considered as a novel method to test the dynamic properties and fatigue behaviors of FAM mixes. Its effectiveness and reliability were demonstrated by multiple tests. The fatigue resistance of FAM mixes could also be greatly influenced by other factors, such as binder grades, asphalt film thickness, RAP sources, aggregate gradation superposition and RA type, which should be taken into consideration in further studies.

## Figures and Tables

**Table 1:** Technical index of virgin asphalt binder.

Technical Index	Unit	Value
Penetration	0.1 mm (25 °C)	68
Softening point	°C	49
Ductility	cm (5 cm/min, 5 °C)	27.8
Viscosity	Pa•s (60 °C)	0.51
Flash point	°C	271
Wax content	%	1.1
Density	g/cm^3^ (15 °C)	1.027

**Table 2:** Summary of FAM mixes. RA = rejuvenating agent.

Mix	Target Binder Content (%)	Binder Replacement Rate (%)	Binder Replacement Content (%)	Virgin Binder Content (%)	RAP Content (%)	RA Content * (%)
0% RAP	9.0	0	0.0	9.0	0.0	-
25% RAP		25	2.2	6.8	29.7	-
50% RAP		50	4.5	4.5	61.6	-
50% RAP + RA		50	4.5	4.5	61.6	10

* By weight of target binder content.

**Table 3:** Parameters of fatigue life prediction models. VECD = viscoelastic continuum damage.

Mix	VECD-Based Fatigue Model Parameters							
	$\left|E_{LVE}^{*}\right|\;(\mathrm{MPa})$	m	α	$S_{f}$	$C_{1}$	$C_{2}$	A	B
0% RAP	1361	0.365	3.743	2735	3.95 × 10^−2^	0.358	5.66 × 10^−4^	7.487
25% RAP	2060	0.301	4.322	1940	1.51 × 10^−2^	0.365	1.42 × 10^−5^	8.647
50% RAP	2705	0.250	5.008	1550	6.11 × 10^−2^	0.413	1.30 × 10^−7^	10.016
50% RAP+RA	1812	0.324	4.085	2225	3.37 × 10^−2^	0.318	5.64 × 10^−5^	8.169

Note: $\left|E_{LVE}^{*}\right|$ is the flexural dynamic modulus in the linear viscoelastic region; m is the slope of dynamic modulus versus loading frequency. α equals to 1/(1+m). *S_f_* is damage intensity at failure point. *C*_1_ and *C*_2_ are regression coefficients. *A* and *B* are fatigue model parameters described in Equations (13) and (14).

**Table 4:** Results of time sweep fatigue test.

Mix	Strain level (%)	Fatigue Life *N_f_*	Standard Deviation	Coefficient of Variation (%)	Fatigue Performance Ranking	
					Measured	Predicted
0% RAP	0.10	22,432	1664	7.4	1	1
	0.09	46,310	3801	8.2		
	0.08	122,571	19,473	15.9		
	0.07	329,224	21,940	6.7		
25% RAP	0.10	5062	740	14.6	3	3
	0.09	11,932	1626	13.6		
	0.08	35,858	2142	6.0		
	0.07	116,548	14,384	12.3		
50% RAP	0.10	3524	440	12.5	4	4
	0.09	15,425	1630	10.6		
	0.08	65,568	4608	7.0		
	0.07	282,052	6405	2.3		
50% RAP + RA	0.10	6049	575	9.5	2	2
	0.09	14,782	736	5.0		
	0.08	64,868	7005	10.8		
	0.07	182,665	22,795	12.5		

**Table 5:** Efficiency comparison between TS and LAS tests.

Test Method	Number of FAM Specimens Required for Each Mix	Average Total Testing Time for Each FAM Mix (h)
Time sweep	12	27
Linear amplitude sweep	3	3
